# Rural–urban migration, illicit drug use and hazardous/harmful drinking in the young Thai population

**DOI:** 10.1111/j.1360-0443.2007.02059.x

**Published:** 2008-01

**Authors:** Tawanchai Jirapramukpitak, Martin Prince, Trudy Harpham

**Affiliations:** 1 Postgraduate Studies Programme, Thammasat University Klong Luang, Pathumthani, Thailand; 2 Section of Epidemiology, Institute of Psychiatry DeCrespigny Park, London, UK; 3 Department of Urban, Environment and Leisure Studies, London South Bank University London, UK

**Keywords:** Alcohol-related disorders, illicit drug use, residential mobility, Thailand, transients and migrants, young adults

## Abstract

**Aims:**

Limited data are available about whether rural–urban migration, often characterized by exposure to urban life stress and a reduction in social network and support, can affect the prevalence of illicit drug use and hazardous/harmful drinking. The purpose of our study was to examine the prevalence of these risky behaviours among Thai young adults and to describe their association between their migration status and these outcomes.

**Design:**

A population-based cross-sectional survey.

**Setting:**

A representative sample of 1052 residents, aged 16–25 years (467 males and 585 females) in a suburban community of Bangkok in 2003 and 2004.

**Measurements:**

(i) Exposures*—* migration (defined as the occasion when a young person born in a more rural area moves for the first time into Greater Bangkok); and (ii) outcomes*—* illicit drug use was assessed with an anonymous self-report adapted from the Diagnostic Interview Schedule (DIS) and hazardous/harmful drinking with Alcohol Use Disorder Identification Test (AUDIT).

**Findings:**

The results showed that 10.9% (82 males and 17 females) had illicit drug use and 24.3% (179 males and 62 females) hazardous and harmful drinking. In multivariate analysis, rural–urban migration was not associated with illicit drug use, whereas hazardous/harmful drinking was associated independently with being late migrants, who moved at the age of 15 or older.

**Conclusions:**

Illicit drug use and hazardous/harmful drinking were common among young Thais. The potential effect of migration on hazardous and harmful drinking identified in this study may be helpful for the design and implementation of preventive measures.

## INTRODUCTION

Rural–urban migration is a major contributor to urbanization in many developing countries. The level of this type of internal migration is increasing in many Asian regions [1]. In the last few decades Thailand has also experienced a dramatic growth in internal migration, especially from rural areas to Bangkok and its vicinity. Between 1991 and 2000 the registered population of Pathumthani Province, the northern part of Bangkok Metropolitan Region (BMR), grew from 412 000 to 677 000, a rate of 5% per year, the highest in Thailand. More than half the local population has migrated during their life-time [2].

Migration to urban areas is an activity undertaken primarily by young adults [3] and characterized by exposure to stressful life events, social difficulties and a reduction in social network and support, with clear potential for deviant behaviours and mental problems [4]. In Thailand, illicit drug use and hazardous and harmful drinking have been identified to be among high priority health issues, which contribute to significant mortality and morbidity among young people [5]. Evidence suggests that the use of illicit drugs, particularly methamphetamine, was reported to be common among Thai adolescents [6], whereas hazardous/harmful drinking was widespread among young adults [7]. However, to date there has been insufficient evidence to support whether migration has such a pathogenic effect. Only a few studies, all conducted in North America, have looked at the relationship between internal relocation and substance and behavioural problems. However, these studies appear to have focused only upon the frequency of moving, rather than rural–urban movement, and the initiation and development of these problems in adolescents [8,9]. The context and pattern of migration in this region is also very different from that in Thailand.

Age at time of migration may also affect the impact of migration. Child migrants may appear to be more vulnerable and perhaps more likely to be affected by migration than adult migrants. In addition to stress and reduced social support caused by migration, less personal control over the decision to move [10], reduced contact with close friends after moving [11–13] and the stress of adapting to a new school and friends [14] may also play an important role. On the other hand, young adult migrants, often moving without parents or family members, may lack the social support and protection of family members against the development of deviant behaviours [15].

In summary, little is known about the prevalence of illicit drug use and hazardous/harmful drinking among young Thai people in the community and about migration status as possible risk factors. We hypothesized that migrants in the city would have a higher risk for illicit drug use and hazardous/harmful drinking, possibly explained by having higher levels of stress and lack of social network and support.

## METHOD

### Sample

A catchment area called Rangsit Municipality, part of Pathumthani Province, was selected. It is located adjacent to the north border of Bangkok and in recent years has been integrated into the metropolis. It has had a rapid increase in population over recent decades. The area was typical of many suburban metropolitan districts, consisting of predominately residential and mixed-use communities.

A sample of 1052 eligible residents, aged 16–25 years, living in Rangsit Municipality was recruited. We first enumerated the catchment area populations by knocking on the doors of all households, identifying young people aged 16–25 years. In the event that there was more than one eligible resident in a given household, we selected one at random to be interviewed using the Kish Grid method [16]. If the selected eligible person was not at home at the time of the first approach for interview, substitution was not permitted. We ensured that repeated visits on at least three occasions were made to interview the selected person in order to reduce non-response.

### Procedure

Six trained interviewers interviewed the selected individuals in their own homes during 2003–04. The main survey instrument consisted of two parts: an interviewer-administered questionnaire and a self-administered questionnaire. The study protocol was approved by the Ethics Committees of Thammasat University and Institute of Psychiatry, King's College London.

### Measures

#### Illicit drug use

Illicit drug use was assessed using an anonymous self-report adapted from the substance use/dependence section of the Diagnostic Interview Schedule (DIS) [17]. A list of drug items was provided, including cannabis, amphetamines, opiates, hallucinogens, ecstasy and solvents, which covered the majority of the illicit drugs used in Thailand [5]. Those who reported the use of any of the listed substance within the previous year were regarded as having a history of illicit drug use. Drug dependency was assessed with five questions enquiring about the frequency of drug use, stated dependence, inability to cut down, need for larger amounts and withdrawal symptoms. This self-report questionnaire was completed by respondents in private and returned in a sealed envelope to the interviewer on-site in order to ensure respondent confidentiality. This was feasible because the illiteracy rates among Thai adults aged 15 and above are very low, 2.8% for men and 6.1% for women [18].

#### Hazardous/harmful drinking

Hazardous/harmful drinking was assessed using the Alcohol Use Disorder Identification Test (AUDIT), a structured and standardized instrument which provides valid and reliable detection of hazardous and harmful use of alcohol in a general population [19]. It has been used in a study of the prevalence of alcohol problems among emergency room patients in Thailand, with sensitivity against a previous or current alcohol-related medical diagnosis of 89% [20].

#### Migration history

Migration history was obtained using the approach developed by Institute for Population and Social Research (IPSR) at Mahidol University [21]. Migration was generally defined as a change in usual residence. In this study, usual residence was simply that address where the respondent had lived for 1 month or more at the time of the interview. We ascertained the life-time history of migration, including the places of birth of the respondent, the destinations for all moves, the duration of each stay, the reason for migration and the age at migration. However, the significant migration event was defined as the occasion when a young person, born in a more rural area, moved for the first time into Greater Bangkok or BMR. The age of 15 was used as a critical age-period as Thai children under this age are more likely to move with their parents or adults, as opposed to older children, who by Thai law are able to obtain a paid job and perform certain official acts [22]. Individuals over 15 are therefore able to move on their own and seek work.

#### Socio-demographic factors

Socio-demographic factors included: (i) respondents' sex, age, marital status, level of education and employment status; (ii) head of household's years of education; and (iii) household assets—ownership of items including refrigerator, TV, stereo, telephone, computer, air conditioner, microwave, washing machine and car. Previous studies in developing countries have argued for and used household asset indices as proxies to measure household socio-economic status (or wealth), rather than monetary measures (such as income or expenditure) [23].

#### Life stress

Life stress was assessed with the List of Threatening Events (LTE) [24], which was used to identify 12 recent stressful life events taken from a longer inventory, the Life Events and Difficulties Schedule (LEDS) [25]. The content validity of the measure was assessed by a local expert panel. All items were agreed upon so that they were relevant to the context of young Thai adults.

#### Childhood adverse experiences

Childhood adverse experiences were screened with anonymous self-administered questionnaires. These covered three categories of child abuse (emotional, physical and sexual). Questions on the three types of abuse were taken directly from the Conflict Tactic Scale (CTS) [26]. The CTS has been used for measuring child abuse and neglect in several studies, with some modifications [27–30]. The CTS questions were translated into Thai and then adapted based upon focus group discussions with local key informants in an earlier stage of the project, which provided guidelines on domains (i.e. emotional, physical and sexual abuse) to be included in the questionnaire and items to be included within each domain. The age of 16 or under was agreed as the critical childhood period of enquiry regarding abusive experiences.

#### Long-term difficulties

Long-term difficulties were assessed with the Social Problems Questionnaire (SPQ) [31], a short self-report questionnaire identifying social problems, difficulties and dissatisfactions. The questionnaire covers housing, occupation, finance, social and leisure activities, child/parent and marital relationships, relationships with relatives, friends, neighbours and work-mates and legal problems. The respondent is classified as having a major problem in a particular domain when he/she indicates marked or severe difficulties on one or more items within the category concerned. The measure was modified to provide adequate coverage of events relevant for children and adolescents including school problems [32].

#### Social network

Social network was assessed with the social network scale of the Close Persons Questionnaire (CPQ) [33], a structured questionnaire assessing three dimensions of social network. The social network items include frequency of contact with relatives and friends and with colleagues, attendance of religious services, membership of/attendance at clubs and social organizations and engagement in voluntary service. These items can be divided into three subscales, namely: isolation scale, network beyond the household scale and household size scale, which were used to assess overall level and different aspects of social network.

### Analytical approach

Statistical analyses were performed with STATA version 8. In univariate analysis, we estimated the prevalence of migrants and odds ratios for their association with the two mental health outcomes. The presence of effect modifiers was investigated by stratifying the estimation of the associations between migration status and each of the main outcomes with all potential confounders including age, gender, marital status and head of household's education using the likelihood ratio test. In multivariate analysis (logistic regression) we estimated the independent associations of the three migratory states with the two mental health outcomes having controlled for the potential confounding effects of other variables, which were associated with each of the outcomes. Also included in the multivariate models were factors hypothetically on the causal pathway between migration and the outcomes. These were level of education, employment status, household asset, life event, social problem, social network and number of childhood abuse category. All the prevalence estimates, univariate and multivariate analyses were weighted back, using STATA SVY commands, to take account of the total number of eligible young people in a given household.

## RESULTS

### Participant characteristics

In all, 3469 of the occupied households were identified. A total of 1080 households contained at least one eligible resident. A total of 1052 eligible people completed interviews successfully. Two people were born overseas. While this was not an a priori exclusion criterion, they could not be classified according to rural–urban migrant status and hence were excluded from the migration analyses. Using the definition of rural–urban migration, 454 people [46.3%, 95% confidence interval (CI) 42.8–49.7] were rural–urban migrants, 13.4% moving from more rural areas into BMR before the age of 15 (early migrants) and 32.9% moved into BMR at the age of 15 or older (late migrants).

The socio-demographic characteristics of the non-migrant, early migrant and late migrant groups are compared in Table 1. Late migrants were more likely to be older than early migrants and non-migrants. There was no difference in gender composition between the three groups. In our relatively young sample, late migrants were much more likely to be married than were early migrants and non-migrants. Early and late migrants were less well educated than their non-migrant peers, and were much less likely to still be in full-time education and more likely to be working. Late migrants were more likely to be in a lower social position than early migrants and non-migrants, assessed by head of household education level.

**Table 1:** Results of univariate analysis of migration and socio-demographic factors among 1050 young adults in Pathumthani, Thailand 2003–04.

	*Type of migration*				
			
		*Migrant (%)*			
				
*Demographic variables*	*Non-migrant* *(%)* *n* = *596*	*Early* *n* = *141*	*Late* *n* = *313*	*F*	*P*
Age (years)					
16–19	42.9	51.6	31.8	7.20	0.0008
20–25	57.1	48.4	68.2		
Sex					
Male	45.4	45.2	48.3	0.31	0.73
Female	54.6	54.8	51.7		
Marital status					
Married	15.1	19.8	41.8	21.21	<0.0001
Single	84.7	79.7	57.3		
Widowed/separated	0.2	0.5	0.9		
Qualification					
No qualification	0.9	2.3	0.6	3.16	0.0005
Primary (6 years)	6.9	14.8	17.8		
Secondary (9 years)	38.5	45.6	42.0		
Higher secondary (12 years)	37.4	24.0	28.3		
Higher diploma (14 years)	7.5	5.5	4.9		
University	8.8	7.8	6.6		
Employment					
Working	38.6	47.9	73.4	13.02	<0.0001
Inactive (student/housewife)	50.5	42.0	16.7		
Unemployed	10.8	10.2	10.0		
Head of household's education					
None/little	3.3	8.8	1.9	4.68	<0.0001
Primary (4 years)	33.6	41.5	39.5		
Primary (6 years)	8.7	12.9	20.4		
Secondary (9 years)	12.8	16.6	14.8		
Secondary (12 years)	18.6	7.4	12.6		
Higher diploma (14 years)	9.7	5.5	2.8		
University	13.3	7.4	8.1		

There was a very strong association between age at time of migration and reason cited for migration (*F* = 24.19, *P* < 0.0001). The large majority of those migrating at the age of 15 or older moved to find work (64%). Occasionally they migrated for further education (12.0%), and if they moved with family it was generally women accompanying their husbands (12.4%). For those migrating under the age of 15, they moved predominately for family reasons (55.5%), mainly with their parents. Relatively few had moved primarily for work (20.7%) or education (14.3%).

### Illicit drug use and hazardous/harmful drinking

A history of having used illicit drug in the past year was given by 99 (10.9%) of the sample, 82 males (19.7%) and 17 females (3.3%) [odds ratio (OR) 0.1, 95% CI 0.1–0.1–0.3]. The majority of illicit drug users had a history of cannabis use (6.0%). The second popular drug of choice was amphetamine (4.6%). The prevalence of any substance dependency was 6.0%. Two hundred and forty-one (24.3%) were hazardous and harmful drinkers, 179 males (40.6%) and 62 females (10.2%) (OR 0.2, 95% CI 0.1–0.2).

### Migration

Table 2 shows prevalence of illicit drug use and hazardous/harmful drinking by migration status. Although illicit drug use and hazardous/harmful drinking were generally more common in late migrants, there was no clear evidence for a general association between migration status and illicit drug use. However, there was a marginally significant trend towards a higher prevalence of hazardous/harmful drinking among migrants, particularly late migrants (20.9% versus 24.3% versus 29.7%, *P* = 0.047). The gender differential impact of migration on the two outcomes was also examined. There were significant trends towards higher prevalences of both outcomes among migrants but only in the male group, with the chances of having illicit drug use increasing from 15.1% to 18.4% and to 27.1% (test for trend: *P* = 0.02) and hazardous/harmful drinking rising from 33.8% to 44.9% and to 49.6% (test for trend: *P* = 0.008) for non-migrants, early migrants and late migrants, respectively. No such trends were observed in the female group, with the prevalence of illicit drug use ranging from 5.0%, 0.8% and 1.4% (test for trend: *P* = 0.06), and hazardous/harmful drinking from 10.2%, 7.5% and 11.2% (test for trend: *P* = 0.8) for non-migrants, early migrants and late migrants.

**Table 2:** Prevalence of illicit drug use and hazardous/harmful drinking by migration status among 1050 young adults in Pathumthani, Thailand 2003–04.

		*Illicit drug use*		*Hazardous and harmful drinking*	
			
*Migration* *variables*	*n*	*%*	*OR (95% CI)*	*%*	*OR (95% CI)*
Non-migrant	596	9.6	1	20.9	1
Early migrant	141	8.7	0.9 (0.4–1.8)	24.3	1.2 (0.7–2.0)
Late migrant	313	13.8	1.5 (0.9–2.7)	29.7	1.6 (1.1–2.3)

OR: odds ratio; CI: confidence interval.

### Other potential confounders and mediators

A number of socio-demographic characteristics and other correlates were associated with the two outcomes. Illicit drug use was associated significantly with male gender, lower levels of education, being unemployed, lower education for the head of household, having fewer assets, higher number of stressful life events, more current social problems and more child abuse categories. Hazardous and harmful drinking was associated significantly with being older, male gender, employed, having fewer household assets, more stressful life events, greater number of child abuse categories and fewer social networks (Table 3).

**Table 3:** Prevalence of illicit drug use and hazardous/harmful drinking by socio-demographic factors, recent stressful life events, social problems and social networks among 1052 young adults in Pathumthani, Thailand 2003–04.

		*Illicit drug use*		*Hazardous and harmful drinking*	
			
*Variables*	*n*	*Prevalence*	*OR (95% CI)*	*Prevalence*	*OR (95% CI)*
Age (years)					
16–19	449	11.4	1	17.4	1
20–25	603	10.5	0.9 (0.5–1.5)	28.9	1.9 (1.4–2.7)
		*P* = 0.72		*P* = 0.0002	
Sex					
Male	467	19.7	1	40.6	1
Female	585	3.3	0.1 (0.1–0.3)	10.2	0.2 (0.1–0.2)
		*P* < 0.0001		*P* = 0.0001	
Marital status					
Married	238	10.3	1	20.9	1
Single	808	11.1	1.1 (0.6–1.9)	25.3	1.3 (0.9–1.9)
Widowed/separated	6	0	–	37.5	2.3 (0.4–14.2)
		*P* = 0.73		*P* = 0.34	
Education					
9 years or less	558	14.6	1	25.1	1
>9 years	494	6.7	0.4 (0.2–0.8)	23.3	0.9 (0.6–1.3)
		*P* = 0.005		*P* = 0.57	
Employment					
Working	501	12.1	1	32.6	1
Inactive	455	5.8	0.4 (0.3–0.8)	10.8	0.2 (0.2–0.4)
Unemployed	96	23.4	2.2 (1.0–5.4)	32.8	1.0 (0.5–1.9)
		*P* = 0.0005		*P* < 0.0001	
Head of household education					
6 years or less	539	14.3	1	25.8	1
>6 years	513	7.1	0.4 (0.3–0.7)	22.6	0.8 (0.6–1.2)
		*P* = 0.0012		*P* = 0.3	
Household asset					
0–3	227	15.2	1	32.0	1
6–9	825	9.5	0.6 (0.3–1.1)	21.8	0.6 (0.4–0.9)
		*P* = 0.07		*P* = 0.008	
Life events					
0	268	4.7	1	15.0	1
1	271	13.5	3.2 (1.3–7.5)	24.4	1.8 (1.1–3.1)
≥2	513	12.6	2.9 (1.4–6.2)	28.9	2.3 (1.5–3.6)
		*P* = 0.01		*P* = 0.002	
Social problems					
0	610	8.5	1	22.7	1
1 or more	442	13.9	1.7 (1.0–2.9)	26.3	1.2 (0.9–1.7)
		*P* = 0.03		*P* = 0.26	
Number of child abuse category					
0		7.3	1	19.1	1
1		9.0	1.3 (0.7–2.3)	23.6	1.3 (0.9–1.9)
2		21.6	3.5 (1.8–6.6)	37.0	2.5 (1.6–4.0)
3		25.9	4.4 (1.3–14.9)	29.6	1.8 (0.5–5.8)
		*P* < 0.0001		*P* = 0.0004	
Isolation score					
0	185	11.9	1	22.8	1
1	372	7.3	0.6 (0.3–1.1)	28.6	1.4 (0.8–2.2)
2	271	14.8	1.3 (0.6–2.6)	26.1	1.2 (0.7–2.0)
>2	224	10.9	0.9 (0.4–1.9)	16.8	0.7 (0.4–1.2)
		*P* = 0.12		*P* = 0.04	
Network score					
<15	430	9.3	1	16.6	1
≥15	622	12.0	1.3 (0.8–2.3)	29.8	2.1 (1.5–3.0)
		*P* = 0.28		*P* < 0.0001	
Household size					
0	56	16.7	1	38.5	1
1	211	14.0	0.8 (0.3–2.2)	29.3	0.7 (0.3–1.3)
2	785	9.6	0.5 (0.2–1.3)	22.0	0.5 (0.2–0.8)
		*P* = 0.2		*P* = 0.01	

OR: odds ratio; CI: confidence interval.

### Stratified analyses

There is little evidence to support effect modification to any consistent, coherent or statistically significant degree (Tables 4 and 5). The exception is perhaps gender; the association between migration (particularly late migration) and each of the two outcomes is stronger in young males compared with females. However, while the trend is strong for both outcomes, the likelihood ratio test for the interaction term is statistically significant only for illicit drug use (*P* = 0.002) (Table 4). The association between migration and hazardous and harmful drinking is stronger in single compared with married young adults (*P* = 0.03) (Table 5).

**Table 4:** Odds ratios (ORs) for the association between migration and illicit drug use, stratified by potential confounders among young adults in Pathumthani, Thailand 2003–04.*

*Stratification variable crude ORs (95% CI)*	*Stratum I*	*Stratum II*	*Adjusted OR*	*LR test for interaction*	*P*
Age	16–19	20–25			
Early migrant	1.0 (0.4–2.7)	0.7 (0.3–2.0)	0.9 (0.4–1.8)	0.51	0.78
Late migrant	1.4 (0.6–3.4)	1.6 (0.8–3.4)	1.5 (0.9–2.7)		
Sex	Male	Female			
Early migrant	1.3 (0.6–2.8)	0.2 (0.0–1.3)	0.9 (0.4–1.9)	12.17	0.002
Late migrant	2.1 (1.1–3.9)	0.3 (0.1–1.1)	1.5 (0.8–2.7)		
Marital status	Married	Single			
Early migrant	1.4 (0.3–6.4)	0.8 (0.4–1.8)	0.9 (0.4–1.8)	1.10	0.58
Late migrant	1.4 (0.4–4.4)	1.7 (0.8–3.6)	1.6 (0.9–3.0)		
Head of household education	>6 years	≤6 years		3.33	0.19
Early migrant	1.6 (0.6–4.3)	0.6 (0.2–1.5)	0.8 (0.4–1.6)		
Late migrant	1.1 (0.4–2.6)	1.5 (0.7–3.0)	1.3 (0.8–2.4)		

* Reference category is non-migrant. Crude ORs [95% confidence interval (CI)]. Early migrant 0.9 (0.4–1.8), Late migrant 1.5 (0.9–2.7). LR: likelihood ratio.

**Table 5:** Odds ratios (ORs) for the association between migration and hazardous/harmful drinking, stratified by potential confounders among young adults in Pathumthani, Thailand 2003–04.*

*Stratification variable crude ORs (95% CI)*	*Stratum I*	*Stratum II*	*Adjusted OR*	*LR test for interaction*	*P*
Age	16–19	20–25			
Early migrant	1.5 (0.7–3.0)	1.2 (0.6–2.4)	1.3 (0.8–2.1)	0.65	0.72
Late migrant	1.8 (0.9–3.5)	1.4 (0.9–2.2)	1.5 (1.0–2.2)		
Sex	Male	Female			
Early migrant	1.6 (0.8–3.1)	0.7 (0.3–1.8)	1.3 (0.8–2.1)	3.66	0.16
Late migrant	1.9 (1.2–3.2)	1.1 (0.6–2.1)	1.6 (1.1–2.4)		
Marital status	Married	Single			
Early migrant	0.5 (0.1–0.7)	1.5 (0.9–2.5)	1.2 (0.8–2.0)	6.98	0.03
Late migrant	1.0 (0.5–2.1)	2.0 (1.3–3.2)	1.8 (1.2–2.7)		
Head of household education	>6 years	≤6 years		0.39	0.82
Early migrant	1.3 (0.6–2.7)	1.2 (0.6–2.3)	1.2 (0.7–2.0)		
Late migrant	1.4 (0.8–2.5)	1.7 (1.0–2.8)	1.6 (1.1–2.3)		

* Reference category is non-migrant. Crude ORs [95% confidence interval (CI)]. Early migrant 1.2 (0.7–2.0), Late migrant 1.6 (1.1–2.3). LR: likelihood ratio.

### Multivariate analysis (controlling for confounders)

Logistic regression models were developed to test the effect of migration on the two outcomes, adjusting for potential confounders. Potential confounders controlled for in the model testing for an independent association between migration status and illicit drug use (Table 6) included age, sex, years of education and education of head of household. Potential mediators were then further controlled for in the model, consisting of employment status, assets, life events, social problems and numbers of categories of abuse experienced. An interaction between migration status and gender was included in a separate model. The multivariate model without an interaction term shows no significant effect of migration on illicit drug use (OR = 0.9, 95% CI 0.4–1.8 for early migration, OR = 1.5, 95% CI 0.9–2.7 for late migration). The model with an interaction term (migration × sex) shows that among males, being a late migrant appears to be associated with illicit drug use. Its effect size reduces only slightly after adjusting for all potential confounders. The effect becomes insignificant after adjusting for those potential mediators. On the other hand, among females the effect appears to be in the opposite direction, with non-migrants having the highest risk. While the effects of both early and late migration are positive, the effect of late migration remains significant even after adjusting for all potential confounders.

**Table 6:** The odds ratios following logistic regression for the association between migration and illicit drug use, controlling for potential confounders (with and without a migration by gender interaction term) among young adults in Pathumthani, Thailand 2003–04.

			*With migration* × *sex*			
			
	*Without migration* × *sex*		*Men*		*Women*	
			
	Early migrant	Late migrant	Early migrant	Late migrant	Early migrant	Late migrant
Model 1	0.9 (0.4–1.8)	1.5 (0.9–2.7)	1.3 (0.6–2.8)	2.1 (1.1–3.9)	0.1 (0.01–1.2)	0.1 (0.03–0.6)
Model 2	0.9 (0.4–1.8)	1.5 (0.8–2.7)	1.3 (0.6–2.8)	2.1 (1.1–3.9)	0.1 (0.01–1.2)	0.1 (0.03–0.6)
Model 3	0.7 (0.3–1.6)	1.2 (0.7–2.3)	1.2 (0.6–2.7)	2.1 (1.1–4.0)	0.1 (0.01–1.2)	0.1 (0.03–0.6)
Model 4	0.7 (0.3–1.6)	1.2 (0.7–2.3)	1.1 (0.5–2.5)	1.8 (0.9–3.6)	0.1 (0.01–1.1)	0.1 (0.03–0.6)
Model 5	0.6 (0.3–1.2)	1.1 (0.6–2.0)	0.8 (0.3–1.9)	1.7 (0.9–3.4)	0.1 (0.01–1.4)	0.1 (0.03–0.6)

Model 1: main effect of migration. Model 2: main effect of migration, controlling for age, sex. Model 3: main effect of migration, controlling for age, sex, education level and education of head of household. Model 4: main effect of migration, controlling for age, sex, education level, education of head of household, employment status and assets. Model 5: main effect of migration, controlling for age, sex, education level, education of head of household, employment status, assets, life events, social problems and number of abuse categories.

Potential confounders controlled for in the model testing for an independent association between migration status and hazardous/harmful drinking (Table 7) include age, sex, marital status, years of education and education of head of household. The potential mediators include employment status, network outside the household, asset, life event and the number of child abuse categories experienced. A separate model with an interaction between migration status and marital status is also created. The model without an interaction term suggests an association with late migration. After adjustment, the effect was partially confounded by employment status and negatively confounded by social network outside household. The model with an interaction term (migration × marital status) suggests that the effect of migration is stronger, although non-significant, among both early and late migrants of single martial status.

**Table 7:** The odds ratios following logistic regression for hazardous/harmful drinking by migration status, controlling for potential confounders (with and without a migration by marital status interaction term) among young adults in Pathumthani, Thailand 2003–04.

			*With migration* × *sex*			
			
	*Without migration* × *sex*		*Married*		*Single*	
			
	Early migrant	Late migrant	Early migrant	Late migrant	Early migrant	Late migrant
Model 1	1.2 (0.7–2.0)	1.6 (1.1–2.3)	0.5 (0.1–1.7)	1.0 (0.5–2.1)	3.1 (0.8–12.4)	2.1 (0.9–5.0)
Model 2	1.3 (0.8–2.1)	1.5 (1.0–2.2)	0.6 (0.2–2.0)	1.0 (0.5–2.1)	2.7 (0.7–11.2)	2.1 (0.9–5.1)
Model 3	1.3 (0.8–2.3)	1.5 (1.0–2.3)	0.7 (0.2–2.7)	0.9 (0.4–2.1)	2.0 (0.5–8.6)	2.0 (0.8–5.1)
Model 4	1.3 (0.8–2.2)	1.5 (1.0–2.4)	0.7 (0.2–2.7)	0.9 (0.4–2.1)	2.1 (0.5–9.0)	2.0 (0.7–5.3)
Model 5	1.2 (0.7–2.1)	1.2 (0.8–1.9)	0.7 (0.2–2.4)	0.9 (0.4–2.0)	2.1 (0.5–8.6)	1.6 (0.6–4.2)
Model 6	1.3 (0.7–2.2)	1.4 (0.9–2.2)	0.9 (0.2–3.3)	1.0 (0.4–2.3)	1.6 (0.4–7.0)	1.6 (0.6–4.4)
Model 7	1.0 (0.6–1.9)	1.2 (0.8–2.0)	0.7 (0.2–2.4)	0.8 (0.3–1.9)	1.7 (0.4–7.2)	1.8 (0.6–5.1)

Model 1: main effect of migration. Model 2: main effect of migration, controlling for age. Model 3: main effect of migration, controlling for age, sex. Model 4: main effect of migration, controlling for age, sex, marital status, education level and education of head of household. Model 5: main effect of migration, controlling for age, sex, marital status, education level, education of head of household and employment status. Model 6: main effect of migration, controlling for age, sex, marital status, education level, education of head of household, employment status and network outside the household. Model 7: main effect of migration, controlling for age, sex, marital status, education level, education of head of household, employment status, network outside the household, asset, life event and number of abuse categories.

## DISCUSSION

To our knowledge, this is the first community study to examine the prevalence of two common problem behaviours, illicit drug use and hazardous/harmful drinking, and their associations with rural–urban migration among young people. We also examined the potential impact of migration at the defined critical age-period on the two outcomes. Our results suggest that there was no significant risk for illicit drug use among early and late migrants in the univariate model. However, in the stratified analyses strong interactions of migration with gender were identified, with late migrant men having a significantly higher risk than early migrant and non-migrant peers. The effect appears to be in the opposite direction in women, with non-migrant women at significantly greater risk than early or late migrants. The interaction effect remained even after adjusting for all potential confounders.

The reason for the potential gender difference in the effect of migration is still unclear. Only one study in Canada has been conducted previously to investigate the possibility of gender difference in the impact of migration [8], which revealed strong sex differences with statistically significant relationships between moving and early drug use initiation and progression occurring primarily among males, whereas females who reported moving twice were significantly less likely than non-movers to hasten their time to onset of first alcoholic beverage. The gender differential effect in this study needs to be interpreted with caution, however. It was not a primary hypothesis for this investigation, and sample size calculations were therefore not carried out to ensure adequate statistical power. Illicit drug use was much less common among women compared to men, effectively reducing the statistical power to detect associations within the female group. Type I error is therefore a possible alternative explanation.

Hazardous/harmful drinking was the only outcome associated consistently with migration. The association between late migration and alcohol problems remained the most robust, having adjusted for all likely confounders (OR = 1.5, 95% CI 1.0–2.4), although the effect size was rather modest. The effect decreased substantially after adjusting for employment status, which may indicate effect mediation rather than confounding. Late migrants are more likely to be either working or unemployed and much less likely to be economically inactive (housewife and student). The fact that the majority of late migrants moved to seek work may explain this mediating effect. Late migrants probably use alcohol as a means to socialize and establish friendships with peers, or to deal with job-related stress or unemployment frustration [34].

No studies have been conducted to investigate the relationship between rural–urban migration and hazardous and harmful drinking. The available evidence investigates only the impact of geographic relocation and drinking problems and does not generally support our findings. A recent Brazilian study [35] found no significant effect of migration on high-risk drinking among adults aged 20 or above. DeWit [8] found that relationships between moving before the age of 16 and alcohol problems were either weak or insignificant among Canadian young adults aged 18–35 years.

A number of limitations to the study should also be noted. A cross-sectional survey design employed by this study does not permit assessment of direction of causality, because exposure and outcome are ascertained simultaneously. Reverse causality is therefore particularly difficult to exclude as an explanation for the findings. Drug use in male late migrants may have preceded and contributed to the migration event. It is also possible that a common underlying trait, e.g. novelty seeking, might have predisposed both to the substance and alcohol abuse and to the migration. Individuals constantly seeking new and exciting experiences are much more likely to abuse drugs and alcohol than are individuals who have less need for novel stimulation [36].

The associations between migration and mental and behavioural problems are complex. Although this study has suggested some possible links between rural–urban migration and illicit drug use and hazardous/harmful drinking, the relationship may not be a direct one. In identifying priorities for future research, longitudinal data in the study of migration and its effect on these outcomes with a larger sample is recommended. There is also a need to test for possible alternative mechanisms that mediate the effects of moving on these risky behaviours.
